# Artificial Intelligence Clinical Reasoning in Board-Style Clinical Vignettes: A Comparative Study

**DOI:** 10.7759/cureus.94563

**Published:** 2025-10-14

**Authors:** Lorela Gjunkshi, Ledio Gjunkshi, Kenneth A Quezada, Nadiya A Persaud, Joseph Braun

**Affiliations:** 1 College of Medicine, Saba University School of Medicine, The Bottom, NLD; 2 Research, Orlando College of Osteopathic Medicine, Winter Garden, USA

**Keywords:** artificial intelligence, clinical-decision making, clinical reasoning, large language models, medical education

## Abstract

Aim: This study evaluated the diagnostic accuracy of four large language model (LLM) artificial intelligence (AI) platforms in generating primary and differential diagnoses using United States Medical Licensing Examination (USMLE) Step 1 clinical vignettes.

Methods: Ten USMLE Step 1 clinical vignette questions were selected, and answer choices were removed to simulate open-ended diagnostic reasoning. Each LLM-ChatGPT GPT-4o-mini (OpenAI), Meta AI Llama 4, Google Gemini 2.0 Flash, and Claude Sonnet 4 (Anthropic)-was prompted to provide both a primary diagnosis and a ranked differential diagnosis. Responses were evaluated using a three-point scoring rubric: 2 points for a correct final diagnosis, 1 point for a correct differential diagnosis only, and 0 points for an incorrect or missing diagnosis. The total possible score per model was 20 points.

Results: Claude Sonnet 4 achieved the highest accuracy with a total score of 20/20 (100%), followed by Google Gemini at 19/20 (95%), ChatGPT GPT-4o-mini at 17/20 (85%), and Meta AI Llama 4 at 13/20 (65%). All models demonstrated clinically relevant reasoning; however, diagnostic prioritization and accuracy varied by platform.

Discussion: The findings indicate that current LLMs possess strong potential as supplemental tools for diagnostic reasoning and medical education. Their ability to generate accurate diagnoses from complex clinical scenarios suggests value for training and clinical decision support. However, variability across platforms highlights the need for cautious implementation. Ethical considerations-including algorithmic bias, overreliance on AI-generated outputs, and patient privacy-must be addressed prior to clinical integration. Future research should incorporate larger and more diverse case sets, include specialty-specific assessments, and establish governance frameworks to guide responsible AI use in medical settings.

## Introduction

Artificial intelligence (AI)

Artificial Intelligence (AI) is defined as a computer that can perform tasks typically done by humans. This includes decision making, reasoning, and the ability to create [1-3]. The origins of artificial intelligence can be traced to Alan Turing's foundational work in 1950, with the term “artificial intelligence” subsequently coined by John McCarthy as the field developed [4,5]. During this period, AI was primarily applied to mathematical computations, including advanced, multifactorial calculations such as determining rocket trajectories [6]. Convinced that AI held greater potential, Turing sought to test the limits of his creation and explore whether AI could truly think beyond its predetermined programming. This pursuit led to the development of the Turing test, which involved three participants: (1) a human judge, (2) an AI interlocutor, and (3) a human interlocutor [6,7]. The human judge interacted with both the AI interlocutor and the human interlocutor through a computer interface, without knowing which one they were communicating with [7]. The human judge was then provided with a set of open-ended and personal questions to pose to both the AI and human interlocutor [7]. The judge then attempted to determine, based on the responses, whether they were interacting with an AI interlocutor or a human [5]. An AI interlocutor was considered to have passed the Turing test if (1) the judge mistakes it for a human, or (2) the judge cannot reliably distinguish its responses from those of a human [6,7]. The Turing test was a landmark in history, establishing a foundational basis for the continued growth and development of artificial intelligence.

Machine learning

Following the development of artificial intelligence (AI), the evolution of AI led to the creation of machine learning (ML). Machine Learning is defined as “AI systems that learn from historical data” [3]. In 1952, Arthur Samuel developed one of the first examples of machine learning through the game of checkers and subsequently coined the term “machine learning” [8]. While machine learning falls under the broader umbrella of AI, it represents a distinct subset of the field. In 1957, Frank Rosenblatt advanced machine learning by building on Samuel’s terminology and applying a brain cell interaction model originally proposed by Donald Hebb [8]. This machine, named the “Mark I Perceptron,” was a machine learning system designed for image and visual pattern recognition [8]. While promising, the machine struggled to recognize complex patterns, such as faces, which ultimately led to the discontinuation of the research [8].

Large language models (LLMs)

Large language models (LLMs) are a category of artificial intelligence (AI) designed to understand and generate text, with the goal of mimicking human language in their responses. Similar to other AI models, LLMs can learn and adapt based on the data used to train them [9,10]. Many widely used AI models are classified as LLMs, including ChatGPT, Google Gemini, Meta Llama, Claude, and others. Because these models are open source, they are easily accessible to the general public. The speed and accessibility of LLMs have led individuals to adopt them for day-to-day tasks and problem-solving, where they have proven both helpful and time-saving.

Using LLMs in medical education

LLMs have many applications, one of which is in the context of medical education. Given the vast amount of information students must master in medical school, having a tool that can access the open web and deliver results within seconds represents a significant advancement in educational innovation and technology. LLMs are adaptive systems capable of reasoning and generating analyses on a variety of topics [10-12]. In this study, LLMs will be used to evaluate the clinical reasoning skills commonly required of medical students and practicing physicians.

In medical education, a variety of national standardized examinations are used to assess students’ clinical reasoning. The most important include the United States Medical Licensing Examination (USMLE) Step 1, Step 2, and Step 3 for students pursuing an allopathic medical degree (MD), and the Comprehensive Osteopathic Medical Licensing Examination of the United States (COMLEX-USA) Level 1, Level 2, and Level 3 for students pursuing an osteopathic medical degree (DO) [13-15]. This study will focus on USMLE Step 1 questions, evaluating the ability of an LLM to apply clinical reasoning in formulating a diagnosis.

Study objective

The objective of this study is to evaluate and compare the diagnostic accuracy of four large language models (ChatGPT, Google Gemini, Claude, and Meta AI) in formulating primary and differential diagnoses using USMLE Step 1 clinical vignettes. The goal is to determine how reliably each model applies clinical reasoning principles and to identify key differences in performance that may inform their potential use in medical education and training.

By presenting a direct comparison of LLMs using standardized medical questions, this study aims to provide clear, evidence-based insights into their diagnostic capabilities, limitations, and implications for integration into medical curricula.

## Materials and methods

This study evaluated four large language models (LLMs): ChatGPT (GPT-4o-mini), Meta AI (Llama 4), Google Gemini (2.0 Flash), and Claude (Sonnet 4). To minimize bias, only the free versions of these LLMs were utilized. Each model was prompted with the same questions verbatim. Using the publicly available USMLE resources, each LLM was prompted with a USMLE Step 1 clinical vignette-style question, with the multiple-choice answer options removed. Each LLM was asked to generate a final diagnosis and a differential diagnosis based on the case presentation. The responses were then compared to the correct answer provided by the USMLE. 

Sample selection

A total of ten questions were selected from the publicly available USMLE Step 1 sample question set, provided as a downloadable PDF on the official USMLE website [14,15]. Questions were selected using a stratified approach to ensure diversity in medical disciplines and complexity levels. Specifically, two questions were drawn from each of the cardiovascular, endocrine, gastrointestinal, neurologic, and infectious disease systems to provide a representative sample of Step 1 content domains. Only questions requiring diagnostic reasoning rather than recall of isolated facts were included. Questions with image-based components or numerical answer options were excluded to maintain consistency across text-only prompts.

Procedures

Each LLM was accessed via its publicly available web-based interface using a standard browser (Google Chrome, Version 128.0.6613.120, Google, Mountain View, California). Prior to each session, browser cache and cookies were cleared, and a new chat window was opened to eliminate contextual memory carryover from prior prompts. Each question was entered exactly as presented in the USMLE source, excluding answer options, followed by the standardized prompt: “What is the most likely diagnosis? What condition(s) should be on the differential, and why were they ruled out?” Models were restricted to their default settings with no plug-ins or external browsing capabilities enabled, ensuring outputs reflected each model’s intrinsic reasoning ability. Responses were collected sequentially and recorded in a Google spreadsheet for analysis.

Data analysis

**Figure 1:**
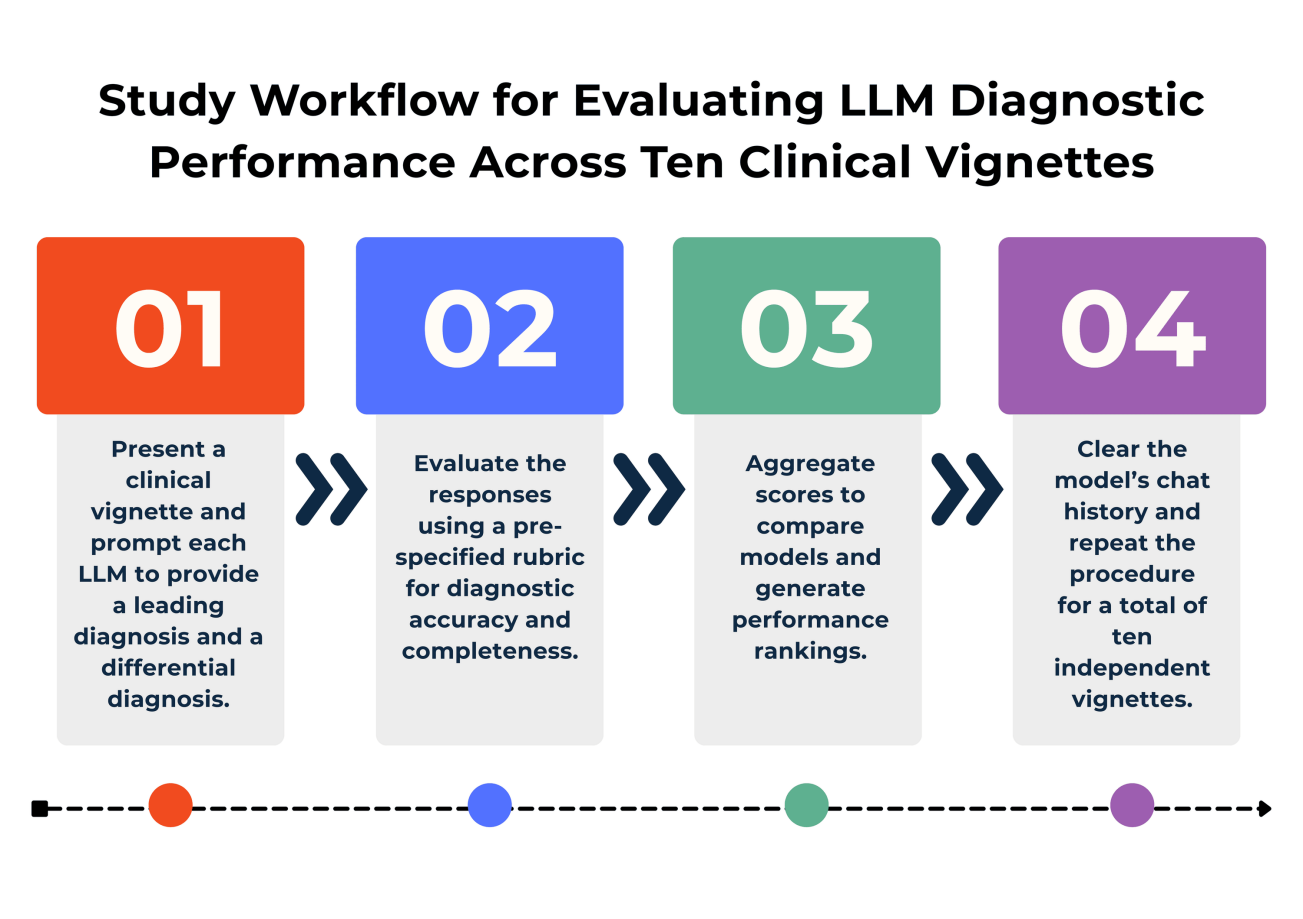
Schematic representation of study methodology. LLM: large language model. Infographic designed by Nadiya A. Persaud on Canva. Software: Canva Pro (Canva Inc., Perth, Australia).

Responses were independently evaluated by two reviewers trained in medical education and Step 1-style assessment. Each answer was scored using a predefined three-point rubric: 2 points if the correct diagnosis appeared as the final diagnosis, 1 point if the correct diagnosis appeared only in the differential, and 0 points if the correct diagnosis was absent entirely. Discrepancies between reviewers were resolved by consensus after joint review of the model output. Aggregate scores were calculated for each LLM, and total accuracy was expressed as both absolute score (out of 20) and percentage accuracy. Descriptive statistics, including mean scores and error counts, were used to summarize model performance. No inferential statistics were applied due to the small sample size. Figure 1 provides a schematic overview of the study design, illustrating the stepwise workflow from question selection through LLM prompting, scoring, and data recording.

## Results

Each large language model (LLM) was individually presented with the same series of ten USMLE Step 1 clinical vignettes, using an identical prompt for each question. Each response was recorded in a spreadsheet, where the LLMs were scored based on the accuracy of their diagnoses. Based on the final scoring of all of the LLMs, the results revealed that Claude Sonnet 4 demonstrated the highest performance, followed by Google Gemini 2.0 Flash, ChatGPT GPT-4o-mini, and Meta AI Llama 4. 

**Figure 2:**
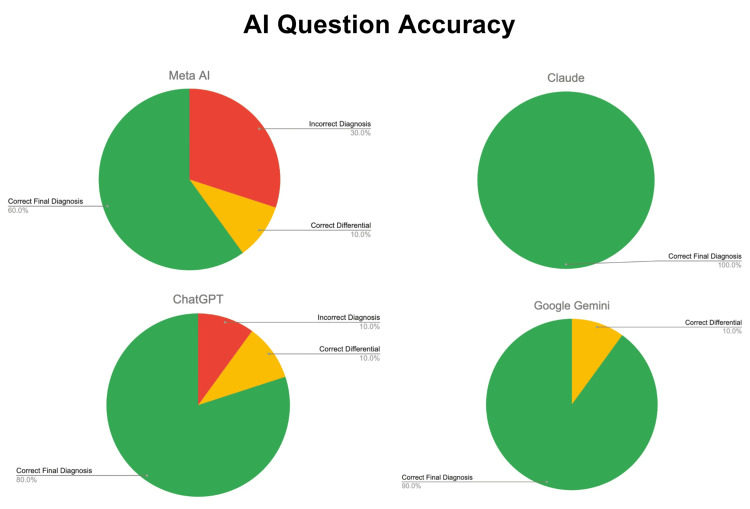
Accuracy of LLM performance on USMLE Step 1 clinical vignettes. Green indicates full points awarded; yellow indicates partial credit (one point); red indicates no points awarded. LLM: large language model; USMLE: United States Medical Licensing Examination. Chart designed by Nadiya A. Persaud on Canva. Software: Canva Pro (Canva Inc., Perth, Australia).

The performance breakdown of each LLM yielded varying results. Claude Sonnet 4 answered all ten clinical vignettes correctly, earning the maximum score allotted in this study. Google Gemini 2.0 Flash had one point deducted for listing the correct diagnosis in its differential but not as its final diagnosis. ChatGPT GPT-4o-mini lost a total of three points: two for failing to include the correct diagnosis in both the final and differential lists on the final or differential list, and one for listing the correct diagnosis in the differential but not as the final answer. Meta AI Llama 4 had seven points deducted: four for missing the correct diagnosis in both the final and differential lists on two vignettes, two for refusing to answer one vignette, and one for including the correct diagnosis in the differential but not as the final diagnosis. These results were compiled and presented in a pie chart to reflect the overall performance of each model (Figure 2).

## Discussion

Differences between large language models (LLMs) 

The results indicate that, in terms of clinical reasoning accuracy, Claude performed the best, followed by Google Gemini, ChatGPT, and lastly Meta AI. One notable finding was that Meta AI refused to answer one of the clinical vignettes. Even after refreshing and opening a new window, the model continued to decline responding to that specific case.

Claude's strong performance across all ten vignettes highlights its consistency and alignment with the level of clinical reasoning expected on standardized exams such as the USMLE Step 1. In comparison, Google Gemini performed well overall but lost points when the correct diagnosis appeared only in the differential rather than as the final answer, indicating a subtle yet important lapse in prioritization and clinical judgment. ChatGPT GPT-4.o-mini demonstrated greater variability, missing some diagnoses entirely while at times identifying the correct diagnosis but failing to prioritize it appropriately. Meta AI Llama 4 showed the weakest performance, with multiple missed diagnoses, a refusal to answer one vignette, and inconsistent prioritization of correct answers.

While the majority of responses were clinically coherent, several LLMs generated inaccurate yet persuasive explanations for incorrect diagnoses. These represent examples of AI hallucinations, where false or misleading information is presented with apparent confidence and supporting rationale. Such occurrences were observed most frequently in cases where models provided detailed pathophysiologic reasoning that appeared plausible but did not align with the correct diagnosis. Although no evidence of algorithmic bias or exposure of sensitive information was detected in this dataset, the presence of hallucinations underscores a key limitation of current LLMs in clinical reasoning tasks. These findings reinforce the importance of critical human oversight and verification when interpreting AI-generated content.

From a medical education standpoint, these results provide practical guidance for medical educators seeking to integrate LLMs safely into training. LLMs can be used as supplemental tools to enhance diagnostic reasoning exercises, simulate clinical vignettes, or support learning, provided that appropriate safeguards are in place. Educators should encourage students to use LLMs as a supplementary tool rather than a primary source, requiring verification of outputs against peer-reviewed or textbook references. Faculty oversight should be incorporated to help students identify instances of hallucinations, incomplete reasoning, or bias.

Limitations

There are several limitations of this study that should be noted. The first concerns the subscription status of each large language model (LLM). Only free, publicly available versions were evaluated. It is plausible that results may differ with paid versions of these LLMs, which are marketed as more advanced. Another limitation is the small sample size, as only ten questions were used. A larger question set would have provided more robust data to assess whether one LLM consistently outperformed its counterparts. Additionally, the questions were drawn from a downloadable PDF on the United States Medical Licensing Examination (USMLE) website. This study cannot confirm whether the LLMs accessed this source directly, raising the possibility that correct answers may have been influenced by prior exposure to the material.

Artificial intelligence (AI) hallucinations

A possible explanation for the incorrect diagnoses generated by certain LLMs is the phenomenon of AI hallucinations. This occurs when an LLM produces a false or inaccurate output while presenting supporting data that makes the response appear credible. Such hallucinations represent a critical limitation of LLMs, as they necessitate users to verify information carefully, given the potential for misleading or incorrect outputs [7].

Although hallucinations were identified in the form of confidently stated but incorrect explanations, no instances of algorithmic bias or privacy violations were observed. Future research should quantify the frequency and context of these hallucinations, assess for systematic bias across patient demographics, and develop validation frameworks to mitigate these risks before clinical implementation.

## Conclusions

This study provides insight into the application of artificial intelligence (AI) for clinical reasoning and evaluates the diagnostic accuracy of different large language models (LLMs). As medicine continues to evolve, open-source models offer opportunities to train diverse tools tailored to both physicians and patients, with particular value in resource-limited settings such as rural medicine. This study also highlights the importance of verifying the accuracy of information provided by open-source LLMs. While this information was easily accessible and quickly provided, several instances in this study showed that certain LLMs were inaccurate in their clinical reasoning. This demonstrates that although AI is becoming more advanced, it has not yet reached the stage where it can be relied upon as a primary resource. Further research is needed to define the strengths and weaknesses of LLMs and to guide their adaptation for medical education and clinical practice. Educators can leverage large language models as interactive teaching tools to enhance critical thinking, diagnostic reasoning, and clinical discussion. However, their use should be guided by clearly defined ethical policies, verification protocols, and curricular boundaries to ensure responsible application. Structured integration of LLMs within case-based or problem-based learning environments, under faculty supervision, can optimize educational benefit while preserving academic and clinical integrity. The importance of this lies in the evolving landscape of medicine, the necessity of keeping pace with technological advances, and the responsibility to provide patients with the best possible care.

## Appendices

Clinical vignettes provided to LLMs for diagnostic evaluation

1. A 50-year-old man comes to the office because of a two-month history of increasing daytime somnolence. He has obstructive sleep apnea for which he has only intermittently used a continuous positive airway pressure device. He is 170 cm (5 ft 7 in) tall and weighs 181 kg (400 lb); BMI is 63 kg/m^2^. His temperature is 37°C (98.6°F), pulse is 100/min, respirations are 12/min, and blood pressure is 135/80 mm Hg. Physical examination shows a gray-blue tinge to the lips, earlobes, and nail beds. Cardiac examination shows no other abnormalities. Arterial blood gas analysis on room air shows a pH of 7.31, PCO_2_ of 70 mm Hg, and PO_2_ of 50 mm Hg.

2. A 32-year-old man comes to the office because of a one-day history of cough productive of small amounts of blood and a two-day history of shortness of breath and swelling of his ankles. He also has a two-week history of progressive fatigue and episodes of dark urine. He has no history of major medical illness and takes no medications. His temperature is 37°C (98.6°F), pulse is 90/min, respirations are 18/min, and blood pressure is 175/110 mm Hg. Pulse oximetry on room air shows an oxygen saturation of 91%. Diffuse inspiratory crackles are heard over all lung bases. There is 2+ pitting edema of both ankles. Results of laboratory studies are shown: Hemoglobin, Hematocrit, Serum, Urea nitrogen, Creatinine, Urine RBC: 8.9 g/dL, 27%, 55 mg/dL, 2.9 mg/dL, 20-40/hpf. Urinalysis also shows some dysmorphic RBCs and rare RBC casts. Examination of a kidney biopsy specimen shows crescentic glomerulonephritis and linear deposition of IgG along the glomerular capillaries.

3. A 32-year-old man comes to the office because of a two-week history of fever and throat pain. He is 173 cm (5 ft 8 in) tall and weighs 63 kg (140 lb); BMI is 21 kg/m^2^. His pulse is 110/min, respirations are 16/min, and blood pressure is 98/68 mm Hg. Physical examination shows scattered 2- to 4-cm lymph nodes in the neck, axillae, and inguinal regions. There is a bilateral tonsillar exudate but no ulcerations. Results of laboratory studies are shown: Hemoglobin, Hematocrit, Leukocyte count, Platelet count: 9.6 g/dL, 29%, 1500/mm^3^, 60,000/mm^3^. A heterophile antibody test result is negative.

4. A 60-year-old man comes to the office because of weakness, tingling of his hands and feet, irritability, and forgetfulness for four months. Physical examination shows pallor, weakness, and spasticity. Deep tendon reflexes are increased. Sensation to vibration is absent in the lower extremities. Laboratory studies show megaloblastic anemia, serum anti-parietal cell antibodies, and increased serum concentrations of methylmalonic acid and total homocyst(e)ine.

5. A 26-year-old man comes to the office because of a one-week history of increased urinary frequency accompanied by excessive thirst. He says he has been urinating hourly. Physical examination shows no abnormalities. Serum chemistry studies are within the reference ranges. Urine osmolality is 50 mOsmol/kg H_2_O. After administration of ADH (vasopressin), his urine osmolality is within the reference range.

6. A 53-year-old man comes to the physician because of a dry scaly rash on his body for the past year. He has had a 15 kg (33-lb) weight loss during the past year. He is 178 cm (5 ft 10 in) tall and now weighs 54 kg (120 lb); BMI is 17 kg/m^2^. His stools have a large volume and float.

7. A 39-year-old man comes to the physician because of a six-month history of progressive shortness of breath. He has had a cough productive of white sputum for two years. He smoked one pack of cigarettes daily for 16 years but quit 10 years ago. He is in mild respiratory distress with pursed lips and a barrel chest; he is using the accessory muscles of respiration. Breath sounds are distant, and crackles are present in the lower lung fields bilaterally. Pulmonary function tests show a decreased FEV1:FVC ratio, increased residual volume, and decreased diffusion capacity. An x-ray of the chest shows hyperinflation and hypertranslucency of the lower lobes of both lungs.

8. A previously healthy 33-year-old woman is brought to the emergency department by the Secret Service for stalking the president of the USA for two months. She claims to be married to the president's twin brother and states that the president just had his twin kidnapped to avoid competition. She speaks rapidly and is difficult to interrupt. Her associations are often loose. She says, "I haven't slept for days, but I won't even try to sleep until my husband is rescued. God has been instructing me to take over the White House. I can't wait to be reunited with my husband. I hear his voice telling me what to do." When asked about drug use, she says she uses only natural substances. She refuses to permit blood or urine tests, saying, "I don't have time to wait for the results."

9. A 34-year-old man comes to the office because of a one-month history of diarrhea. He has a history of pheochromocytoma treated two years ago. His mother is being treated for a tumor of her parathyroid gland. He has no other history of major medical illness and takes no medications. His temperature is 37.0°C (98.6°F), pulse is 84/min, respirations are 10/min, and blood pressure is 120/75 mm Hg. Pulse oximetry on room air shows an oxygen saturation of 97%. Vital signs are within normal limits. Physical examination shows a 3-cm, palpable mass on the right side of the neck. A biopsy specimen of the mass shows a neuroendocrine neoplasm of parafollicular cell origin.

10. A 30-year-old woman comes to the office because of a four-day history of an increasingly severe, painful rash over her body and in her mouth. The rash began over her trunk area but spread within a day to her face and extremities. Two days before the development of the rash, she had flu-like symptoms with muscle aches and fatigue, as well as a nonproductive cough, sore throat, and runny nose. Ten days ago, she began treatment with trimethoprim-sulfamethoxazole for a urinary tract infection; she takes no other medications. Temperature is 39.0°C (102.2°F), pulse is 120/min, respirations are 25/min, and blood pressure is 165/105 mm Hg. Physical examination shows diffuse brownish-red macular exanthema with bullous lesions. Epidermis at an uninvolved site can be removed with mild tangential pressure. Examination of a biopsy specimen of one of the lesions shows necrosis of keratinocytes throughout the epidermis. There is minimal lymphocytic infiltration within the superficial dermis.
